# Clinical phenotype of ASD-associated *DYRK1A* haploinsufficiency

**DOI:** 10.1186/s13229-017-0173-5

**Published:** 2017-10-05

**Authors:** Rachel K. Earl, Tychele N. Turner, Heather C. Mefford, Caitlin M. Hudac, Jennifer Gerdts, Evan E. Eichler, Raphael A. Bernier

**Affiliations:** 1 Department of Psychiatry and Behavioral Sciences, University of Washington, CHDD Box 357920, Seattle, WA 98195 USA; 2 Department of Genome Sciences, University of Washington, Seattle, WA USA; 3 School of Medicine, University of Washington, Seattle, WA USA; 4 Howard Hughes Medical Institute, Seattle, WA USA; 5 Center on Human Development and Disability, University of Washington, Seattle, WA USA

**Keywords:** Autism, *DYRK1A*, Genetic syndrome, Genetically defined subtype, Disruptive mutation, Clinical phenotype

## Abstract

**Background:**

*DYRK1A* is a gene recurrently disrupted in 0.1–0.5% of the ASD population. A growing number of case reports with *DYRK1A* haploinsufficiency exhibit common phenotypic features including microcephaly, intellectual disability, speech delay, and facial dysmorphisms.

**Methods:**

Phenotypic information from previously published *DYRK1A* cases (*n* = 51) and participants in an ongoing study at the University of Washington (UW, *n* = 10) were compiled. Frequencies of recurrent phenotypic features in this population were compared to features observed in a large sample with idiopathic ASD from the Simons Simplex Collection (*n* = 1981). UW *DYRK1A* cases were further characterized quantitatively and compared to a randomly subsampled set of idiopathic ASD cases matched on age and gender (*n* = 10) and to cases with an ASD-associated disruptive mutation to *CHD8* (*n* = 12). Contribution of familial genetic background to clinical heterogeneity was assessed by comparing head circumference, IQ, and ASD-related symptoms of UW *DYRK1A* cases to their unaffected parents.

**Results:**

*DYRK1A* haploinsufficiency results in a common phenotypic profile including intellectual disability, speech and motor difficulties, microcephaly, feeding difficulties, and vision abnormalities. Eighty-nine percent of *DYRK1A* cases ascertained for ASD presented with a constellation of five or more of these symptoms. When compared quantitatively, *DYRK1A* cases presented with significantly lower IQ and adaptive functioning compared to idiopathic cases and significantly smaller head size compared to both idiopathic and *CHD8* cases. Phenotypic variability in parental head circumference, IQ, and ASD-related symptoms corresponded to observed variability in affected child phenotype.

**Conclusions:**

Results confirm a core clinical phenotype for *DYRK1A* disruptions, with a combination of features that is distinct from idiopathic ASD. Cases with *DYRK1A* mutations are also distinguishable from disruptive mutations to *CHD8* by head size. Measurable, quantitative characterization of *DYRK1A* haploinsufficiency illuminates clinical variability, which may be, in part, due to familial genetic background.

**Electronic supplementary material:**

The online version of this article (10.1186/s13229-017-0173-5) contains supplementary material, which is available to authorized users.

## Background

Autism spectrum disorder (ASD) is characterized by tremendous clinical variability and causal heterogeneity. Historical efforts to behaviorally subtype ASD have been largely unsuccessful due to lack of meaningful treatment implications by subtype and inadequate consensus regarding clinical phenotype [1, 2]. Recent efforts have targeted the genetic causes of ASD to explore biologically defined subtypes [3, 4]. Advances in genetic sequencing technology have improved our ability to identify disease-causing mutations [5]. Chromosomal abnormalities, copy number variants (CNVs), and disruptive single nucleotide variants (SNVs), including nonsense, frameshift, and splice site mutations, have been associated with increased risk of ASD [6–9]. Most recently, work relating ASD risk to de novo disruptive SNVs suggests these single point mutations account for approximately 10% of ASD cases [6, 8]. These discoveries have prompted a shift in ASD research; instead of using extensive phenotyping prior to sequencing ASD populations, researchers have begun by identifying genes of interest in affected individuals and then exploring phenotype in specific gene cohorts [10].

The application of this genetics-first approach to subtyping ASD has successfully identified similar medical, behavioral, and dysmorphic features shared by individuals with disruptive variants in high-confidence ASD risk genes, such as *CHD8*, *ADNP*, *SCN2A*, and *DYRK1A* (e.g., [11–13]). Dual-specificity tyrosine phosphorylation-regulated kinase 1A, or *DYRK1A*, is a highly conserved gene in the Down syndrome critical region of chromosome 21 [6, 14], and appears to play a major role in brain development, specifically neurogenesis, neural plasticity, and cellular death [15]. *DYRK1A* haploinsufficiency was initially identified for its role in intellectual disability, which is clinically defined as childhood onset of significant cognitive and adaptive impairment [16]. Recurrent disruptions to *DYRK1A* have been found in as many as 0.5% of cases with ASD [14, 17]. In *Drosophila* models, truncating mutations to *DYRK1A* (*Drosophila* ortholog termed the Minibrain (*Mnb*) gene) result in microcephaly, including intact but smaller brain structures [18]. *Dyrk1A*-null mouse models (−/−) displayed growth deficiencies resulting in mid-gestational death [19]. Mice heterozygous for *Dyrk1A* (+/−) survived to adulthood but presented with reduced growth, developmental delays, motor and learning difficulties, and atypical behaviors, including anxiety [19, 20]. A consistent clinical phenotype appears in humans. Reported cases to date have exhibited microcephaly and intellectual disability; other features, including seizures, speech and motor delays, feeding difficulties, and distinct facial dysmorphology, have been noted as well [13, 15, 21–24].

Studies relying on medical record review have reported ASD diagnoses in up to 40% of cases with *DYRK1A* mutations, but many remaining cases have features consistent with ASD, such as stereotypies, reduced eye contact, and social anxiety [15, 21]. Few studies have conducted diagnostic evaluations of ASD and ASD-related symptoms as part of a clinical phenotyping battery. Diagnostic rates may be as high as 88% when ASD is directly evaluated as part of the study assessment process [13].

Literature on *DYRK1A* haploinsufficiency supports its association with ASD risk and suggests a complex phenotype that includes distinct dysmorphology as well as cognitive, neurological, and medical impairments. However, studies to date have only reported categorical descriptions of phenotype, which note the presence or absence of a common phenotype, such as ASD or no ASD. Quantitative assessments of ASD-related features in large cohorts and in relation to other ASD cohorts have not been examined. While previously published reports have noted an emerging phenotypic profile, variability in clinical presentation remains. Measurable data on medical, developmental, and behavioral characteristics are needed to better understand small variations in phenotype between individuals. Furthermore, varied clinical presentations of individuals with *DYRK1A* mutations have yet to be examined in the context of their familial phenotypic profile as a measure of remaining genetic background. This approach has been applied to other developmental disorders and to CNVs associated with ASD, but has yet to be applied to disruptive SNVs associated with ASD [25–28].

The aim of the proposed study was to examine a large cohort of cases with *DYRK1A* mutations, provide a summary of phenotype, and compare recurrent medical and behavioral features to (1) large idiopathic ASD samples and (2) a cohort with disruptive mutations to a different ASD-associated gene, *CHD8*. Alongside *DYRK1A*, *CHD8* is one of the most recurrent genes with disruptive SNVs implicated in ASD and provides a comparison group ascertained in the same way as the cases with *DYRK1A* mutations in this sample [6, 8]. Detection of phenotypic differences between these two groups could inform understanding of different biological profiles of ASD and illuminate key features unique to each disrupted gene. This study also explored the contribution of genetic background to phenotypic variability among individuals with disruptive *DYRK1A* mutations.

## Methods

### Participants

#### *DYRK1A* sample

**Fig. 1:**
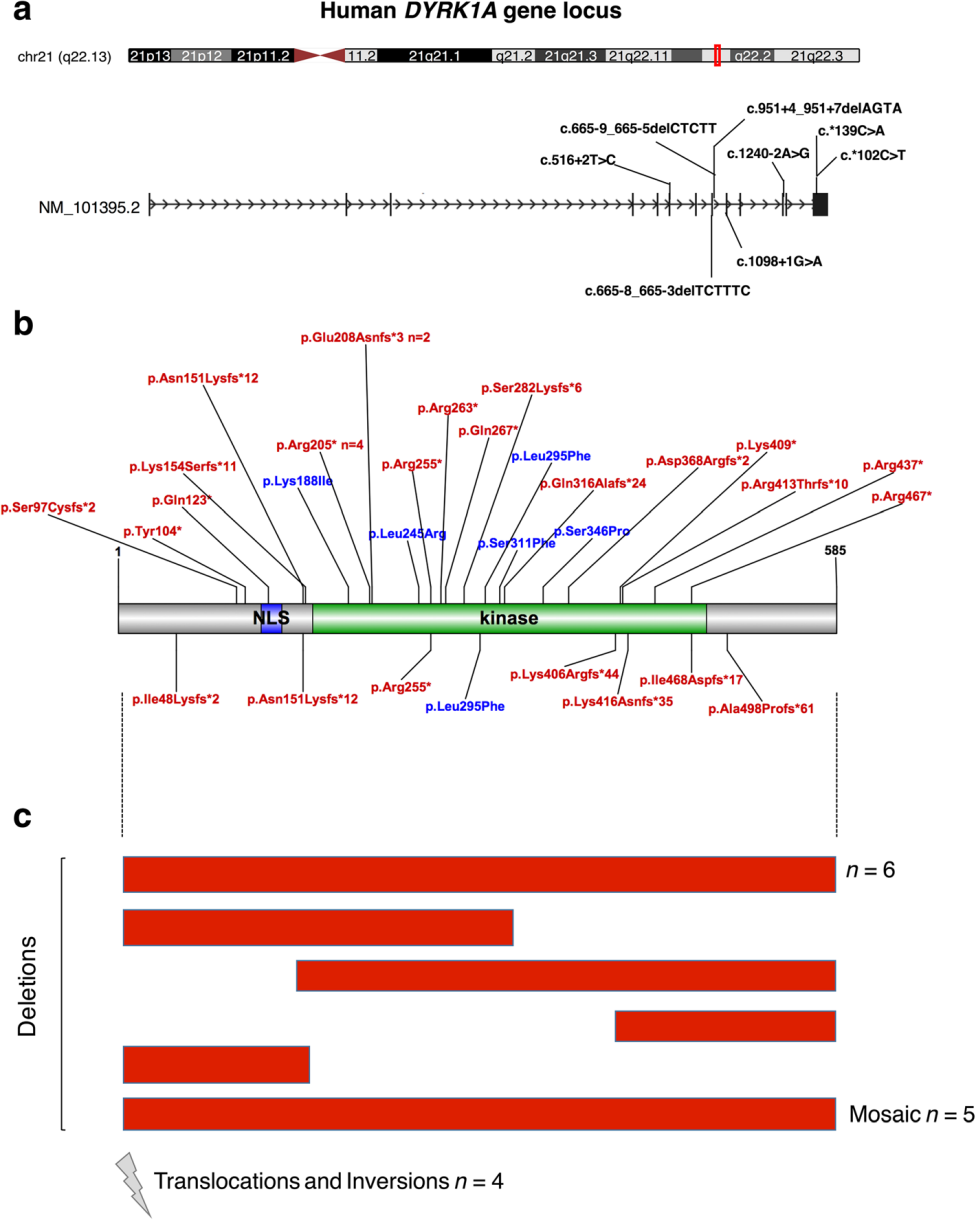
Summary of *DYRK1A* gene variants. Schematic depicting the locations of disruptive variants (truncating, missense, and splice site mutations), copy number variations, and chromosomal rearrangements affecting *DYRK1A*. The ideogram of human chromosome 21 and isoform NM101395.2 coding sequence was obtained from the UCSC genome browser [54]. **a** NM101395.2 coding sequence with eight reported splice site mutations (presented in HGVS cDNA notation). Mutations below the sequence are UW-SNV participants, above are Pub-SNV mutation cases. **b** The *DYRK1A* protein (NP_567824.1) with truncating (red) and missense (blue) mutations (presented in HGVS notation). Mutations below the protein are UW-SNV cases, above are Pub-SNV mutation cases. **c** Copy number deletions and chromosomal rearrangements, including six deletions of entire gene, four partial deletions, five mosaic deletions, and four translocations/inversions (lightning bolt)

Participants included 42 individuals with de novo, disruptive, pathogenic SNVs (nonsense, splice site, frameshift, and missense mutations) at the *DYRK1A* gene. (Fig. 1; see Additional files 1 and 2 for full variant information). The sample includes 10 individuals assessed as part of an ongoing study at the University of Washington (UW), including 7 new cases identified through clinical genetic testing and 3 previously published cases recruited from the Simons Simplex Collection (see below). In addition to the 3 previously published cases studied at UW, 32 other previously published cases with disruptive SNVs were included in the sample. All subjects were identified via clinical exome sequencing, or exome or targeted sequencing of research cohorts ascertained for a diagnosis of ASD or ID.

Those seen at UW (UW-SNV group; *n* = 9 de novo and *n* = 1 non-maternal; see Table 1 for variant information [29]) completed standardized behavioral measures and medical evaluations by clinicians naïve to gene group membership as part of a study evaluating individuals ages four and older with ASD-associated, disruptive mutations. Biological parents of the participants were also characterized.

**Table 1:** *DYRK1A* variant information for UW-SNV mutation patients

Patient	Position	Type of mutation	cDNA	Protein	Inheritance
1	21:38865466	Splice site	c.1098+1G>A	*–*	De novo
2	21:38845116	Frameshift	c.143_144delTA	p.Ile48Lysfs*2	De novo
3	21:38877833	Frameshift	c.1491delC	p.Ala498Profs*61	De novo
4	21:38868533	Frameshift	c.1217_1220delAGAA	p.Lys406Argfs*44	De novo
5	21:38862575	Nonsense	c.763C>T	p.Arg255*	De novo
6	21:38877746	Frameshift	c.1401delAinsGG	p.Ile468Aspfs*17	De novo
7	21:38853064	Frameshift	c.452dupA	p.Asn151Lysfs*12	Not maternal
8	21:38862695	Missense	c.883C>T	p.Leu295Phe	De novo
9	21:38862463	Splice site	c.665-8_665-3delTCTTTC	–	De novo
10	21:38877590	Frameshift	c.1248delA	p.Lys416Asnfs*35	De novo

Variant information for UW-SNV patients using NCBI reference sequence for *DYRK1A* isoform NM_101395.2, GRCh37 (hg19) build version (Ensembl id: ENST00000338785). This isoform was selected because it was the highest expressing isoform in human tissues in the GTEx database [https://gtexportal.org/home/gene/DYRK1A]) [53]. Patients 1–3 were first identified through the Simons Simplex Collection, patients 4–10 underwent clinical genetic testing prior to research participation. cDNA and protein (NP_567824.1) annotation follows HGVS guidelines

Thirty-two previously published cases of *DYRK1A* disruptive SNVs (Pub-SNV group) included 31 de novo cases and 1 non-maternal case [13, 15, 21, 22, 30–32] with available medical history, physical features, and diagnoses.

Additionally, the phenotype of 19 previously published cases of de novo *DYRK1A* chromosomal rearrangements (Pub-CHR group), including microdeletions and translocations, was described and compared to those with disruptive SNVs [21, 24, 33–39]. See Table 2 for participant characteristics for the 61 total *DYRK1A* sample participants.

**Table 2:** Demographics

	*DYRK1A* sample			SSC idiopathic sample		*CHD8* sample
	Disruptive SNVs		*CHR*	No disruptive SNVs or deleterious CNVs		Disruptive SNVs
	Pub-SNV	UW-SNV	Pub-CHR	Total sample	IQ < 70	
Total *N* (male)	32 (22)	10 (4)	19 (9)	1981 (1705)	487 (407)	12(9)
Mean age in months (SD)	124.12 (128.85)	108.40 (69.12)	102.22 (88.06)	107.66 (42.34)	114.00 (44.00)	148.08 (64.56)

Participant demographics. *SNV* single nucleotide variant, *Pub-SNV* published disruptive SNV cases, *UW-SNV* UW study cases with disruptive SNVs, *Pub-CHR* published chromosomal rearrangement, *CNV* copy number variant. Note that there are three overlapping individuals in the Pub-SNV and UW-SNV groups ascertained from the Simons Simplex Collection. DYRK1A sample significantly differed from idiopathic ASD samples (total and IQ < 70) in gender ratio, χ^2^ (1, *n* = 2042) = 66.88, *p* < 0.001 and χ^2^ (1, *n* = 548) = 36.25, *p* < 0.001, respectively. Samples did not significantly differ in age, *p* > 0.05. No significant differences in age or gender for *DYRK1A* and *CHD8* samples

#### Comparison samples

Secondary data from an idiopathic subset of the Simons Simplex Collection (SSC), a large sample ascertained for ASD, were compared to the *DYRK1A* sample. The SSC was a cohort of 2446 simplex families including a single proband with ASD age 4–18, unaffected biological parents, and any unaffected siblings [40]. Probands were included in the idiopathic subset (*n* = 1981) if they had no known disruptive SNVs or deleterious CNVs, as determined by sequencing efforts by Sanders and colleagues in 2015, and not based on any phenotypic or behavioral profile [8]. In order to account for high rates of ID seen in the *DYRK1A* haploinsufficiency, a subset of the idiopathic group with full-scale IQ below 70 (*n* = 487) served as an additional comparison group. Also, a randomly selected age- and gender-matched subset (*n* = 10) of the full idiopathic sample was compared to the subset of *DYRK1A* cases assessed quantitatively at the UW. As part of the SSC, probands were assessed on measures of neurocognitive functioning, social communication behaviors, motor skills, physical features (e.g., head circumference), and medical history (measures described below).

Twelve individuals with disruptive SNVs at a different high-confidence ASD risk gene, *CHD8* (chromodomain helicase-DNA-binding protein 8), participating in the same UW characterization study and assessed by clinicians naïve to implicated gene disruption, served as a comparison cohort matched on ascertainment approach.

### Measures

#### Categorical assessment of diagnostic history and developmental characteristics

Psychiatric and medical history, developmental milestones, and physiological characteristics were gathered from Pub-SNV and Pub-CHR cases. In addition to published data, supplemental case reports detailing medical history and developmental trajectory were reviewed when available.

For UW-SNV study participants, a structured caregiver interview, adapted from the SSC, was administered to gather information about developmental, psychiatric, and medical history. When caregiver-endorsed diagnoses required additional clarification, medical records were reviewed for confirmation. Using all available information, psychiatric diagnoses were either confirmed or newly diagnosed by a licensed clinical psychologist using the *Diagnostic and Statistical Manual of Mental Disorders*, *5th Edition* (DSM-5) [16]. For research purposes, subjects were given an ASD diagnosis based on clinician observation and parent interview using standardized instruments (gold-standard assessment tools described below). A diagnosis of intellectual disability was given when a subject displayed childhood onset of deficits in both cognitive and adaptive functioning. When subjects were under the age of 5 and had failed to reach cognitive developmental milestones at the time of assessment, a diagnosis of Global Developmental Delay was given. A physical and dysmorphology exam was conducted by a licensed medical geneticist.

### Quantitative assessment of *DYRK1A* UW-SNV (*n* = 10) and *CHD8* (*n* = 12)

#### Head circumference

Occipital frontal head circumference was measured and standardized values calculated using a normative population reference [41].

#### Cognitive functioning

Full-scale IQ was assessed in probands and unaffected parents. Probands ages 4 years, 0 months to 17 years, 11 months were administered the *Differential Abilities Scales*, *2nd Edition* [42]. Probands 18 and older, as well as unaffected parents, were administered the Wechsler Abbreviated Scales of Intelligence [43]. For all assessments, IQ scores were generated using deviation (standard; mean = 100, SD = 15) or ratio scores (mental age equivalent/chronological age × 100). Ratio scores were derived using age equivalence values if standard scores were not possible to calculate due to subject’s level of functioning.

#### Adaptive functioning

Caregivers were administered the *Vineland Adaptive Behavior Scales, 2nd edition* (VABS-2) to measure adaptive functioning across communication, daily living skills, and social domains [44].

#### ASD-specific assessment

Research-reliable clinicians administered the appropriate module of the *Autism Diagnostic Observation Schedule, 2nd Edition* (ADOS-2; [45, 46]) and *Autism Diagnostic Interview-Revised* (ADI-R; [47]). ADOS calibrated severity scores, and items regarding age of first words and age of first steps from the ADI-R were used in analyses. The total *T* score from the Social Responsiveness Scale (SRS-2; [48]) was used to quantify ASD-associated symptoms in all UW-SNV family members.

### Analytic approach

#### Categorical variables

Fisher’s exact tests were used to compare frequencies of features commonly found for *DYRK1A* mutations across disruptive SNV (Pub-SNV and UW-SNV) and chromosomal rearrangement (Pub-CHR) groups. These features included intellectual disability, speech delay (defined as first words after 24 months of age), motor deficits (e.g., delayed walking, poor coordination, abnormal gait), ASD-related deficits (e.g., ASD diagnosis, stereotypic behaviors, anxious behaviors), feeding difficulties, seizures, vision abnormalities, and microcephaly. The frequency of most common features (defined as present in 75% or more cases) was compared across *DYRK1A* and idiopathic ASD groups (total idiopathic sample and subset with IQ below 70). Only characteristics specifically noted in case reports were included in analyses; if a phenotypic characteristic was not reported, it was treated as missing for that individual. Total frequencies reflect cases with a reported presence or absence of a given characteristic.

#### Quantitative variables

UW-SNV *DYRK1A* participants were compared to (1) a randomly subsampled age and gender-matched subset of the SSC idiopathic sample and (2) a cohort with disruptive *CHD8* mutations on domains of functioning assessed quantitatively, including head circumference, IQ, adaptive functioning, ASD severity (ADOS calibrated severity score), age of first words (ADI-R), and age of first independent steps (ADI-R). Independent sample *t* tests were used to compare the *DYRK1A*, idiopathic, and *CHD8* groups, using the Bonferroni adjustment for multiple comparisons (*p* < 0.002).

Nonparametric Wilcoxon signed rank tests were used to compare parental and proband phenotype for UW-SNV participants in head circumference, IQ, and ASD symptoms (SRS). Gene “effect size,” measured as the difference between parental and proband phenotype, was calculated as follows:

$$ \mathrm{Effect}\  \mathrm{size}=\frac{\mathrm{Proband}\  \mathrm{mean}-\mathrm{U}\mathrm{naffected}\  \mathrm{biparental}\  \mathrm{mean}}{\mathrm{Unaffected}\  \mathrm{biparental}\  \mathrm{standard}\  \mathrm{deviation}\ } $$

When both maternal and paternal data were available, biparental means were calculated as the average of maternal and paternal scores. If only one parent’s data was available, that parent’s score was used instead of a biparental mean.

## Results

### Clinical phenotype of *DYRK1A*

There were no significant differences between disruptive SNV (Pub-SNV and UW-SNV) and chromosomal rearrangement (Pub-CHR) groups on frequency of phenotypic characteristics (Table 3). Language delay was noted for 61/61 (100%); 21 individuals were nonverbal at the time of their evaluation. Intellectual disability and/or Global Developmental Delay (depending on age) were reported in 60/61 (98%) cases. The presence of motor difficulties, including delayed walking, abnormal gait, and poor coordination, was noted for 52/53 (98%). A common abnormal gait was observed across UW-SNV participants, specifically a lilting gait with a forward lean to the upper body, arms bent and held tight against the body, and hands splayed. Feeding difficulties in infancy, including poor suck, were observed in 51/54 (94%) of those with reports of feeding abilities in early development. Microcephaly, defined as head circumference two or more standard deviations below the mean for age, either primary (present throughout development) or acquired at a later age, was reported in 58/61 cases (95%). Vision abnormalities were identified in 34/42 (81%) cases, including impairments such as strabismus, astigmatism, optic nerve dysfunction, and corneal clouding. Febrile and non-febrile seizures were reported in 42/58 cases (72%).

**Table 3:** Phenotypic characteristics of *DYRK1A*

	Pub-SNV and UW-SNV (*n* = 42)			Pub-CHR (*n* = 19)			Total (*n* = 61)			
Phenotypic characteristic	*N*	Total	%	*N*	Total	%	*N*	Total	%	Sig (Fisher’s exact tests)
Intellectual disability or Global Developmental Delay	41	42	98	19	19	100	60	61	98	NS
Speech delay	42	42	100	19	19	100	61	61	100	NS
Motor difficulties	38	38	100	14	15	93	52	53	98	NS
Microcephaly	39	42	93	19	19	100	58	61	95	NS
Feeding difficulties	37	40	93	14	14	100	51	54	94	NS
Vision abnormalities	26	33	79	8	9	89	34	42	81	NS
Seizures	26	39	67	16	19	84	42	58	72	NS
ASD diagnosis	16	35	46	2	7	29	18	42	43	NS
Stereotyped behaviors	22	36	61	4	9	44	26	45	58	NS
Anxious behaviors	11	36	31	1	8	13	12	44	27	NS
Hyperactive behaviors	10	35	29	4	8	50	14	43	33	NS
Behavioral differences	35	42	83	7	19	37	42	61	69	NS
6+ symptoms including ASD	25	42	60	7	19	37	32	61	52	NS
6+ symptoms including broader behavioral difficulties	32	42	76	10	19	53	42	61	69	NS

Frequency of phenotypic features in cases with disruptive SNVs (Pub-SNV and UW-SNV) to *DYRK1A*, published chromosomal rearrangements (Pub-CHR) to *DYRK1A*, and total combined cases. Totals reflect those with complete data. Groups did not significantly differ in gender ratio (Fisher’s exact test) or age (independent sample *t* test), *p* > 0.05. Fisher’s exact tests used for group comparisons, *Sig* significance, *NS* not significant

Diagnoses of ASD were reported in 18/42 cases (43%), suggesting elevated risk well above the general population percentage of 1.5% [49]. The frequency increased to 42/61 cases (69%) when broadening the criteria to include ASD-related behaviors without a formal diagnosis, such as stereotypic behaviors (e.g., complex motor mannerisms, repetitive and self-stimulatory behaviors), limited eye contact (reported in those without known severe vision impairments), inappropriate laughter, and limited social engagement. Anxious behaviors were reported in 12/44 cases (27%), and hyperactivity was reported in 14/43 cases (33%). Seven of the ten UW-SNV cases were confirmed to have ASD; three who did not meet diagnostic criteria presented with notable stereotypies and socially anxious behaviors.

Co-occurrence of the seven most common phenotypic features (reported in 75% or more of cases) was evaluated: microcephaly, intellectual impairment, speech delay, motor difficulties, feeding difficulties, vision abnormalities, and ASD. Fifty-two percent of the total *DYRK1A* sample (32/61) possessed six or more features. Sixty-nine percent (42/61) presented with six or more features when the ASD category was broadened to include other behavioral difficulties, including stereotypic, anxious, and hyperactive behaviors.

**Fig. 2:**
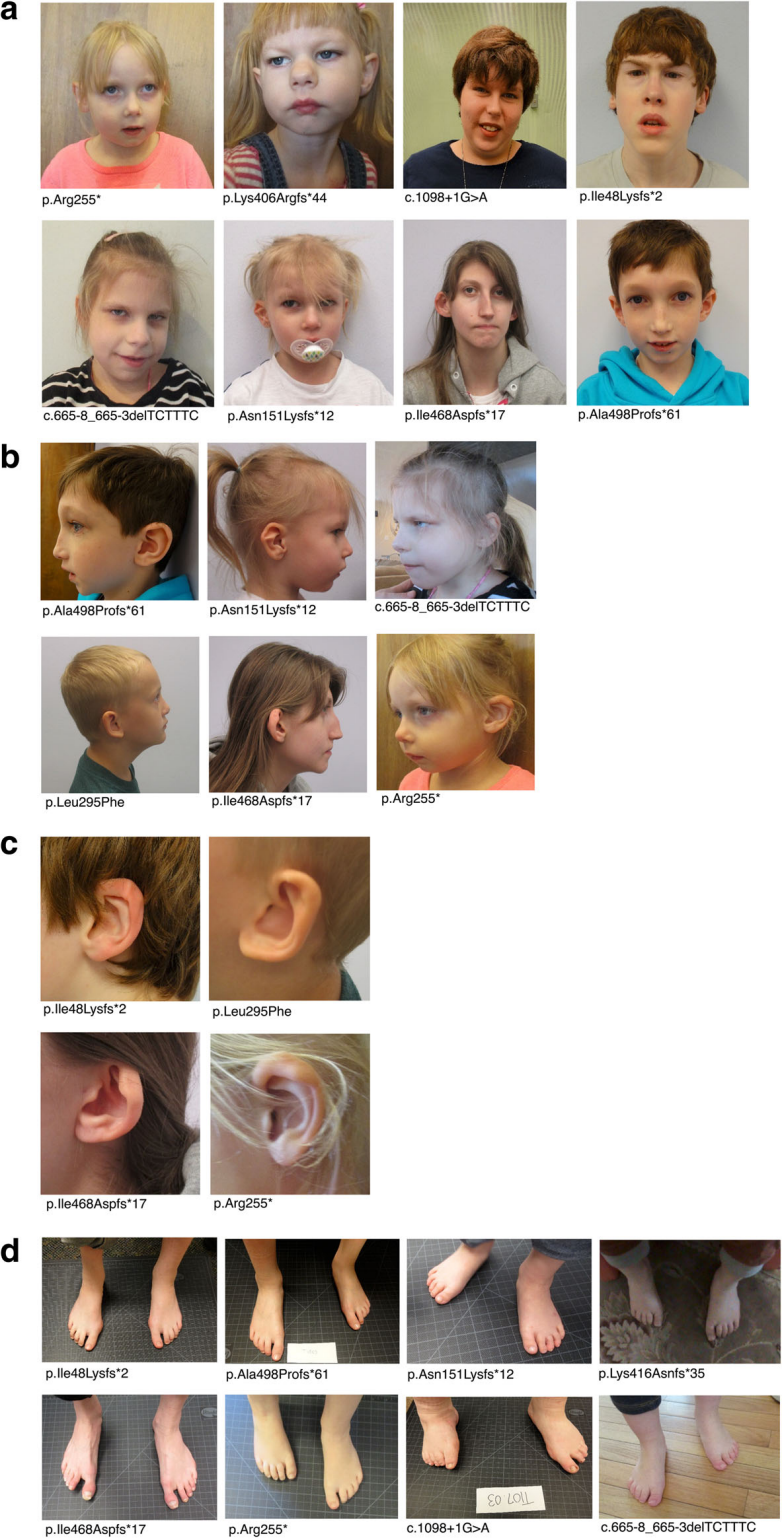
Common dysmorphic features in UW-SNV patients with *DYRK1A* haploinsufficiency. **a** Facial features of eight UW-SNV patients with *DYRK1A* haploinsufficiency. Note common features across patients, including prominent brow with high anterior hairline, slightly upslanted palpebral fissures, retrognathic jaw, deep-set eyes with a hooded appearance, bitemporal narrowing, high nasal bridge with tubular-shaped, broad-tipped nose, and protruding ears. **b** Profiles of six UW-SNV patients. Note prominent brows with high anterior hairlines as well as low-set, posteriorly rotated ears in a subset of patients. **c** Ear abnormalities in four UW-SNV patients, including post-rotated and protruding ears with protruding thick and overfolded helices (i.e., outer fold of the ear). **d** Foot abnormalities in eight UW-SNV patients. Common features include proximal placement of the first toe, crooked toes, and syndactyly of the second and third toes. Frameshift, nonsense, and missense cases identified by HGVS protein notation; cases with splice site variants identified by HGVS cDNA notation

Facial dysmorphisms were reported in 50/51 (98%) previously published cases (excluding UW-SNV cases who were previously published, *n* = 3). Similar dysmorphic facial features were observed in eight UW-SNV cases who participated in a standardized medical exam (five new cases, three previously published), including deep-set eyes with a hooded appearance, slightly upslanting palpebral fissures, bitemporal narrowing, prominent brow with high anterior hairline, tubular-shaped nose, prominent nasal bridge, retrognathic jaw, and small chin (Fig. 2a). Additionally, prominent, low-set, or malformed ears were also reported across cases; 4/8 UW-SNV cases presented with thick, overfolded ear helices (Fig. 2b). Foot anomalies were also noted across patients, including toe syndactyly (webbing of the toes), arachnodactyly, crooked toes, and proximal placement of the first toe (Fig. 2c). Observed commonalities in facial, ear, and foot characteristics in UW-SNV cases were consistent with reports of previously published cases. In the larger sample, spine or chest abnormalities, including pectus excavatum and scoliosis, were reported in 13/25 cases with documented skeletal observations.

### Phenotypic comparisons of *DYRK1A* to idiopathic ASD

**Fig. 3:**
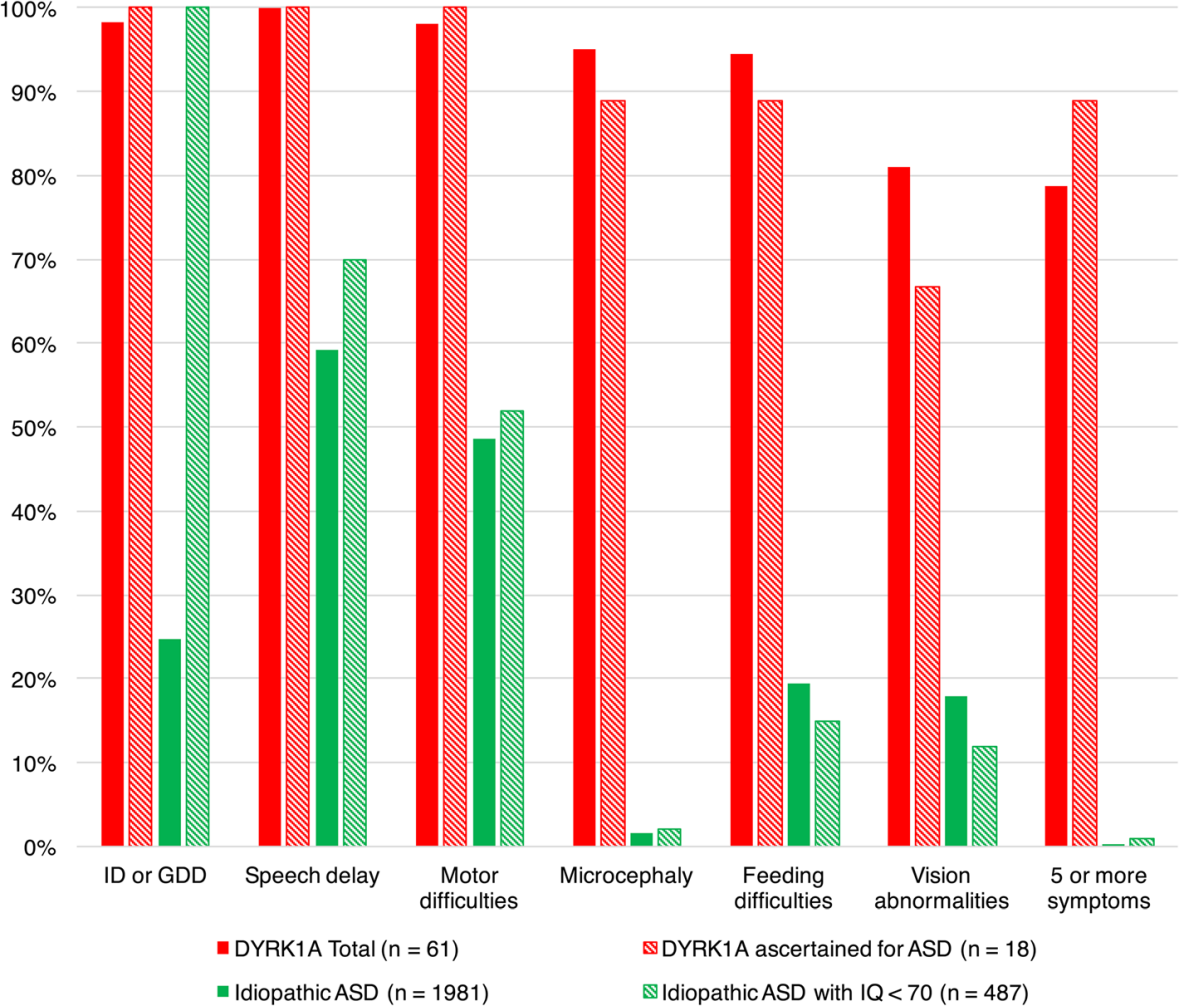
Phenotypic features in total *DYRK1A* sample, *DYRK1A* sample ascertained for ASD, and idiopathic ASD samples. Bar graph presented frequencies of core phenotypic features observed in 75% or more of *DYRK1A* patients. Total *DYRK1A* sample (Pub-SNV, UW-SNV, Pub-CHR) and *DYRK1A* sample ascertained for ASD were compared to frequencies of features in idiopathic ASD samples (total and IQ < 70) using Fisher’s exact tests (*p* < 0.001)

Rates of microcephaly, intellectual disability, speech delay, motor difficulties, vision impairments, and feeding difficulties were significantly higher in the total *DYRK1A* group (Pub-SNV, UW-SNV, and Pub-CHR combined) relative both to the full idiopathic SSC comparison cohort and the subset with IQ below 70 (Table 4; Fig. 3).

**Table 4:** Phenotypic comparisons of *DYRK1A* to idiopathic ASD

	Total *DYRK1A* sample (*n* = 61)		*DYRK1A* ascertained for ASD (*n* = 18)		SSC idiopathic ascertained for ASD (*n* = 1981)		Sig (total DYRK1A vs idio total)	SSC idiopathic ASD with IQ < 70 (*n* = 487)		Sig (total DYRK1A vs idio IQ < 70)
Phenotypic characteristic	*N*/total	%	*N*/total	%	*N*/total	%		*N*	%	
Intellectual disability or Global Developmental Delay	60/61	98	18/18	100	487/1974	25	*p* < 0.001	487/487	100	–
Speech delay	61/61	100	18/18	100	1173/1981	59	*p* < 0.001	343/487	70	*p* < 0.001
Motor difficulties	52/53	98	18/18	100	963/1981	49	*p* < 0.001	253/487	52	*p* < 0.001
Microcephaly	58/61	95	16/18	89	31/1958	2	*p* < 0.001	10/485	2	*p* < 0.001
Feeding difficulties	51/54	94	16/18	89	386/1981	19	*p* < 0.001	112/487	15	*p* < 0.001
Vision abnormalities	34/42	81	16/18	67	355/1981	18	*p* < 0.001	60/487	12	*p* < 0.001
5+ symptoms	48/61	79	16/18	89	6/1981	0.30	–	5/487	1	–

Frequency of core phenotypic features (included if reported in 75% or more cases) observed in total *DYRK1A* sample (Pub-SNV, UW-SNV, and Pub-CHR) compared to frequency of same features in those with *DYRK1A* mutations ascertained for ASD, a large sample of cases with idiopathic ASD from the Simons Simplex Collection (ascertained for ASD), and a subset of idiopathic cases with IQ < 70. Totals reflect those with complete data. Fisher’s exact tests used to compare total *DYRK1A* sample to both idiopathic samples on each phenotypic characteristic; all group differences significant, *p* < 0.001. *Sig* significance

The majority of the *DYRK1A* group (79%) displayed five or more of these phenotypic features in combination. The percentages and group differences remain in the subset of individuals with *DYRK1A* mutations ascertained for an ASD diagnosis (*n* = 18, Fig. 3). Co-occurrence of five or more features increased to 89% in those with *DYRK1A* mutations ascertained for ASD.

### Quantitative phenotype of *DYRK1A*

#### *DYRK1A*vs*idiopathic ASD*

**Fig. 4:**
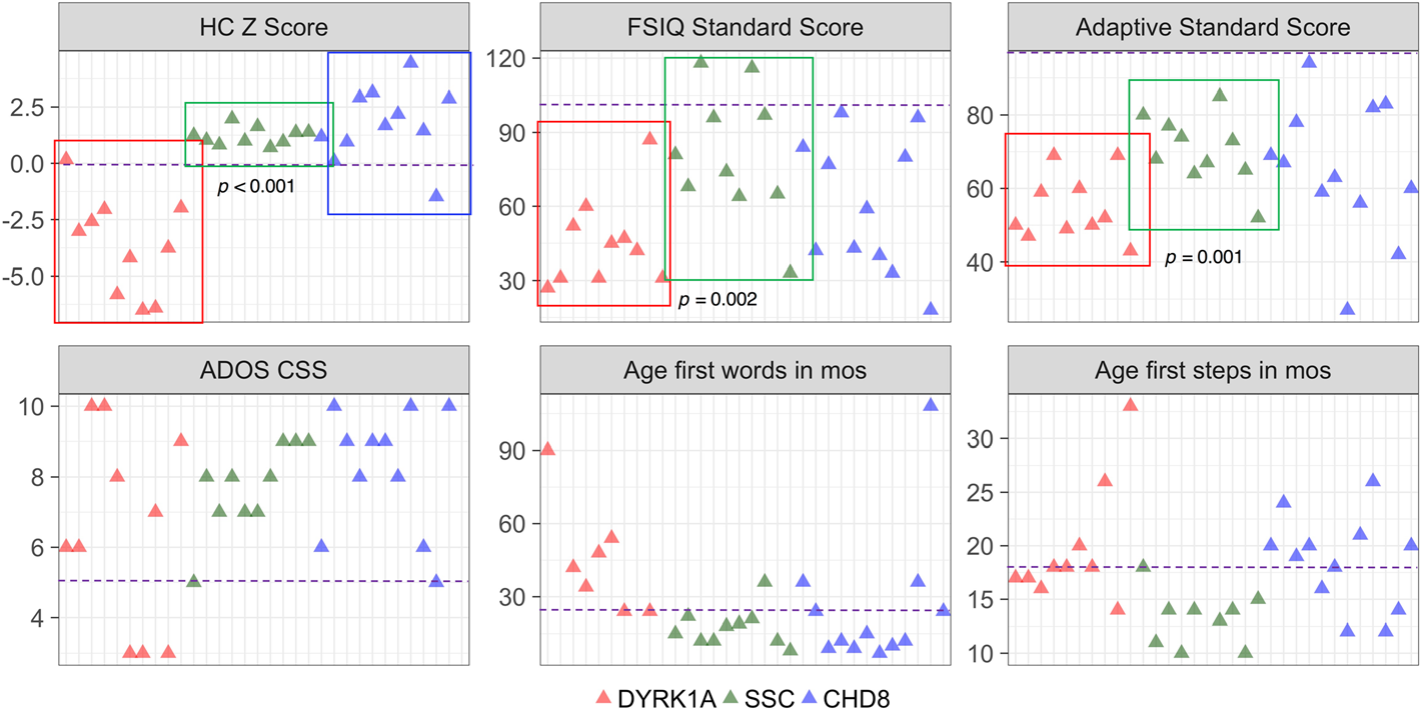
Quantitative phenotype of *DYRK1A*, idiopathic, and *CHD8* samples. Scatterplots of core phenotypic features in UW-SNV *DYRK1A* sample, (*n* = 10), idiopathic subset matched for age and gender (randomly sample, *n* = 10), and *CHD8* sample (*n* = 12). Dotted lines designate conservative averages for typical population. *HC* head circumference, *FSIQ* full-scale IQ, *ADOS CSS* calibrated severity score. Independent sample *t* tests comparing *DYRK1A*, idiopathic, and *CHD8* groups, *p* value adjusted for multiple comparisons

Independent sample *t* tests revealed significant differences between UW-SNV and a matched idiopathic group on measures of head circumference, cognitive ability, and adaptive functioning (Table 5; Fig. 4). The UW-SNV group had significantly smaller head circumference (*p* < 0.001), significantly lower full-scale IQ (*p* = 0.002), and significantly lower adaptive abilities (*p* = 0.001) compared to the idiopathic group. There were no differences between groups on autism symptom severity (ADOS calibrated severity score), age of first words, or age of first independent steps. Outliers across the six phenotypic features of interest represented different UW-SNV individuals, highlighting the variability that is observed when exploring the phenotype quantitatively.

**Table 5:** Quantitative phenotype and group differences between *DYRK1A*, idiopathic, and *CHD8* groups

	*DYRK1A* (UW-SNV)			SSC idiopathic subset			*CHD8*			*t* statistics *DYRK1A* vs SSC		*t* statistics *DYRK1A* vs *CHD8*		*t* statistics *CHD8* vs SSC	
Phenotypic characteristic	Mean	SD	*N*	Mean	SD	*N*	Mean	SD	*N*	*t*	*d*	*t*	*d*	*t*	*d*
Head circumference *Z* score	− 3.62	2.17	10	1.21	0.39	10	1.76	2.9	11	6.94*	3.10	6.49**	2.84	NS	–
Full-scale IQ	45.30	18.14	10	81.20	26.12	10	60.91	27.39	11	3.57*	1.60	NS	–	NS	–
Overall adaptive functioning	54.80	9.04	10	70.50	9.40	10	65.00	18.49	12	3.81*	1.70	NS	–	NS	–
Autism severity (ADOS CSS)	6.50	2.80	10	7.70	1.25	10	8.18	1.78	11	NS	–	NS	–	NS	–
Age walked unaided	19.70	5.64	10	13.22	2.59	10	18.50	4.38	12	NS	–	NS	–	NS	–
Age of first single words	45.14	22.82	7	17.50	7.92	10	25.17	28.01	12	NS	–	NS	–	NS	–

Descriptives for phenotypic variables commonly impaired in *DYRK1A* haploinsufficiency for three groups: *DYRK1A* (*n* = 10), SSC idiopathic subset randomly samples and matched on age and gender (*n* = 10), and *CHD8* (*n* = 12). Group comparisons using independent sample *t* tests between (1) *DYRK1A* and SSC groups, (2) *DYRK1A* and *CHD8* groups, and (3) *CHD8* and SSC groups. *Significant differences between *DYRK1A* and idiopathic groups, *p* < 0.002; **Significant differences between *DYRK1A* and *CHD8* groups, *p* < 0.001. Independent sample *t* and Cohen’s *d* values provided when significant, *p* value adjusted for multiple comparisons, *NS* not significant

#### *DYRK1A*vs*CHD8*

No significant differences were found between *CHD8* and idiopathic groups. However, UW-SNV and *CHD8* groups differed significantly in head circumference (*p* < 0.001), such that *DYRK1A* cases had significantly smaller head size than *CHD8* cases. IQ, adaptive functioning, autism symptom severity, age of first words, and age of first steps were similar across groups (Table 5; Fig. 4).

### Contribution of genetic background

**Fig. 5:**
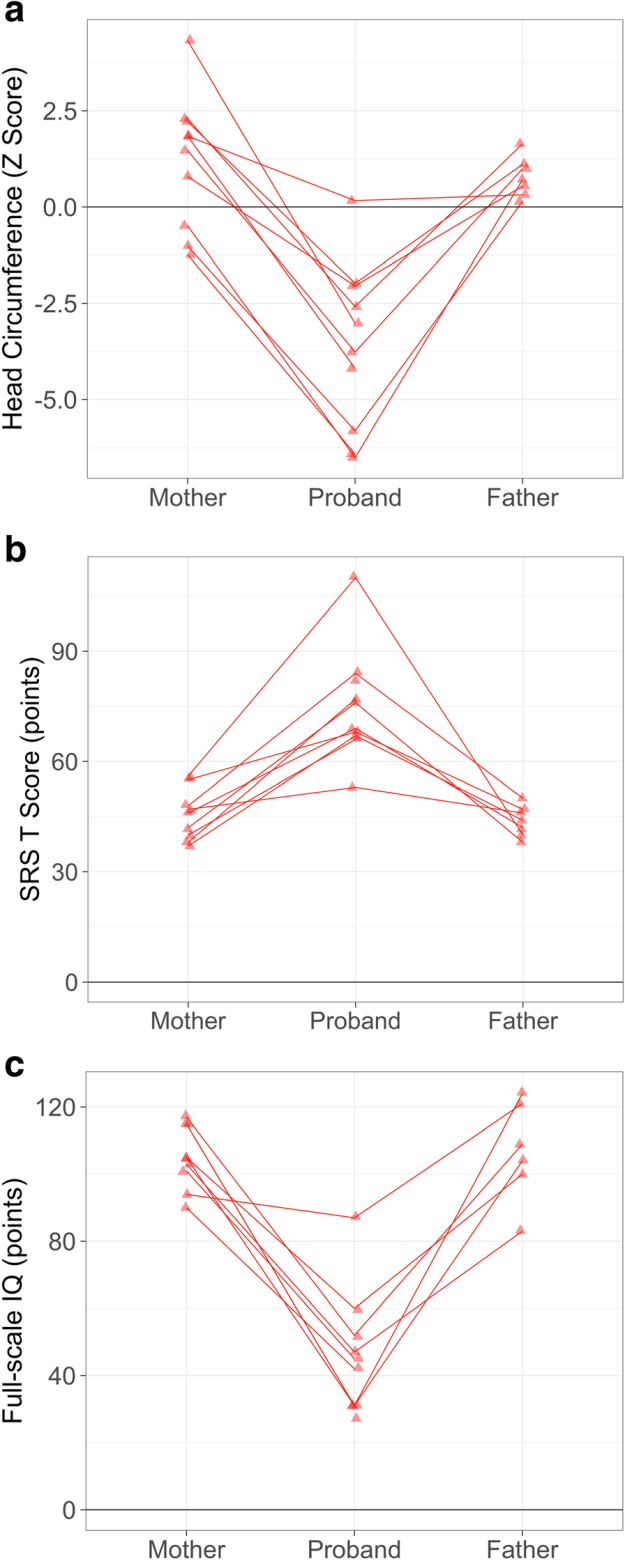
Contribution of familial genetic background to head circumference, ASD symptoms, and IQ. UW-SNV cases are presented with their unaffected mothers and fathers on three phenotypic measures: **a** head circumference (*Z* score, SD), **b** ASD symptoms (Social Responsiveness Scale *T* score), and **c** IQ (full-scale standard score). Affected children presented with significantly more severe phenotypes compared to both unaffected mothers and fathers using Wilcoxon rank sum tests (*p* < 0.001). Variability in parental phenotype corresponds to proband variation. Probands with smaller head sizes relative to other UW-SNV cases correspond to parents who also have smaller head size and vice versa. There are similar patterns in cognition, perhaps more pronounced for fathers, such that fathers with higher IQ have probands with higher IQ relative to other *DYRK1A* cases. Related to social responsiveness, higher parental scores (i.e., greater social impairment) correspond to probands with greater social impairment. Also, note the apparent wider range of IQ variability for fathers (*SD* = 14.99) relative to mothers (*SD* = 9.42) and the wider range of head circumference variability for mothers (*SD* = 1.81) relative to fathers (*SD* = 0.52)

When comparing parental and proband head circumference *Z* score, the presence of a *DYRK1A* mutation accounted for a 2.93 SD decrease in head size for probands. Wilcoxon signed rank tests showed that both mothers and fathers exhibited significantly larger head size, controlling for age and gender, compared to their affected child (*Z* = −2.67, *p* = 0.008 and *Z* = −2.20, *p* = 0.028, respectively (Fig. 5a)). When comparing ASD-related symptoms, *DYRK1A* accounted for a 5.51 SD increase in SRS total *T* score (more symptoms). Wilcoxon signed rank tests showed that both mothers and fathers display significantly lower SRS scores compared to their affected child (*Z* = −3.62, *p* < 0.001 and *Z* = −3.41, *p* = 0.001, respectively (Fig. 5b)). On measures of full-scale IQ, *DYRK1A* accounted for a 6.09 SD decrease in IQ for probands compared to biparental IQ. Wilcoxon signed rank tests showed that both mothers and fathers display significantly higher IQ compared to their affected child (*Z* = −2.67, *p* = 0.008 and *Z* = −2.20, *p* = 0.028, respectively (Fig. 5c)).

## Discussion

This study of the *DYRK1A* haploinsufficiency phenotype, compiling previously published and newly identified cases, confirms a phenotype characterized by microcephaly, intellectual disability, speech delay, motor difficulties, feeding difficulties, and vision abnormalities. A common facial gestalt included deep-set eyes with a hooded appearance, slightly upslanted palpebral fissures, tubular-shaped nose with pronounced broad tip, high nasal bridge, prominent brow with high anterior hairline, retrognathic jaw, and small chin. Dysmorphic feet, including proximal placement of the first toe, syndactyly of the second and third toe, and unusually long and/or crooked toes, and protruding, post-rotated ears with overfolded, thick helices were also commonly observed. Those with de novo disruptive SNVs and chromosomal rearrangements did not differ in clinical features.

Of those case studies where ASD was mentioned and/or evaluated, 43% of probands received an ASD diagnosis. Among 15 cases who received gold-standard ASD assessment, rates increased to 73%. Additionally, features common to ASD, such as stereotypic and anxious behaviors, were noted in many cases where reference to an ASD diagnosis was absent. This suggests rates of ASD in *DYRK1A* cohorts may be higher in reality than reported in the total sample of *DYRK1A* cases published to date.

There are several reasons for the potential underestimated prevalence rate of ASD among published *DYRK1A* cases. First, most previously published cases relied on medical records, which varied greatly in the detail provided and discussion of comprehensiveness of prior evaluations; as such, it is unknown whether ASD was either evaluated and ruled out, or not evaluated at all. Second, it can be difficult to tease apart symptoms of ASD from those of intellectual disability and speech impairments without specialized training and experience with differential diagnosis within developmental disabilities, particularly in children with complex medical histories. Additionally, establishing an ASD diagnosis may not be the most pressing concern for families (and perhaps providers) given the array of impairments and medical conditions that often accompany children with a *DYRK1A* mutation. As *DYRK1A* haploinsufficiency continues to be explored within ASD risk, these factors need to be considered when determining rates in this population.

In an effort to situate the *DYRK1A* phenotype in the context of ASD, we found the *DYRK1A* group (Pub-SNV, UW-SNV, and Pub-CHR groups combined) exhibited significantly higher incidence of key features compared to those with idiopathic ASD: intellectual disability, speech delay, motor difficulties, vision abnormalities, feeding difficulties, and microcephaly. Frequency of these features also significantly differed between the *DYRK1A* group and the comparison group with idiopathic ASD and IQ below 70. This is consistent with prior evidence that disruptive SNVs and CNVs often result in significantly more impairing comorbidities than in idiopathic ASD [6, 8]. Notably, when those with *DYRK1A* mutations who were originally ascertained for an ASD diagnosis were compared to the idiopathic group (also ascertained for ASD), the profile remains the same. This provides further support that the phenotype commonly exhibited in individuals with *DYRK1A* disruptions and ASD is indeed distinct from idiopathic ASD. The co-occurrence of five or more of these phenotypic features in *DYRK1A* cases (79% of total sample, 89% of those ascertained for ASD) provides support for further exploration of *DYRK1A* haploinsufficiency in an individual presenting with concerns of ASD and this combination of phenotypic features.

Prior publications of *DYRK1A* mutation cases have relied on categorical data to describe clinical phenotype. Our exploration of a quantitative phenotype suggested that *DYRK1A* haploinsufficiency is differentiable from idiopathic ASD by measures of cognition, adaptive skills, and head size and distinguishable from a different ASD-associated gene mutation, *CHD8* by head size. It is possible that further phenotypic differences exist which have not been detected by current diagnostic tools given limits to the level of resolution inherent in clinical assessment. Markers relying on quantitative, brain-based measures may reveal gene-specific profiles. For instance, recent work highlights divergent information processing systems for children with 16p11.2 CNVs [50] and children with an early-emerging disruptive SNV [51]. Considering the intellectual disability associated with *DYRK1A* haploinsufficiency, a passive, noninvasive neuroimaging approach may help illuminate neuroendophenotypes that link the behavioral phenotype to the underlying neural mechanisms.

Exploring quantitative phenotype in UW-SNV participants illuminated phenotypic heterogeneity among individuals. While *DYRK1A* mutations significantly impact functioning in a number of domains, the severity of impairment varied among individuals. Family background may, in part, contribute to this variability. While still exploratory, variability in parental phenotype corresponded with variability observed in probands with *DYRK1A* haploinsufficiency. Most striking were familial patterns on measures of head circumference. Even with the range of microcephaly, probands with the smallest head sizes were related to parents with smaller head sizes compared to other parents within the UW-SNV group. Physiological characteristics are among the most highly correlated between parents and children in typically developing populations, ranging from 0.5 to 0.7 [52, 53]. Our findings suggest that, even in the presence of a de novo, disruptive *DYRK1A* mutation, parental phenotype may still impact their affected child’s presentation. Of course, secondary genetic events, embryonic or early developmental influences, and treatment must also be considered as potential factors contributing to the variability.

Our findings must be considered in the context of limitations of this study. First, information available for previously published cases varied widely. While some case reports provided detailed record of psychiatric history, others only included medical history, which also varied in its extensiveness. Full assessment history was unknown for previously published cases, raising questions whether phenotypic features left out of a case report were previously ruled out and confirmed absent or were not assessed. These variations highlight the importance of consistency in phenotypic assessment across future *DYRK1A* phenotype studies to ensure comprehensive and accurate phenotyping efforts. Second, those with *DYRK1A* mutations who participated in the same quantitative assessment battery remain small in number. Larger sample sizes are indeed needed to better understand the quantitative phenotype of *DYRK1A* haploinsufficiency and potential variability between affected individuals. Also, while comparison of *DYRK1A* mutation cases to idiopathic ASD provides important confirmation of distinct comorbidities within ASD, it is important to acknowledge that individuals in the idiopathic group may, with future advances in our understanding of the genetics of ASD, no longer be identified as idiopathic. The idiopathic group analyzed in this study likely represents a population with fewer syndromic features than populations with ASD and other genetic events. Thus, further studies and larger samples of other ASD-associated gene mutations are needed to further distinguish how the *DYRK1A* haploinsufficiency phenotype differs from that of other disruptive gene events. Future studies of this population should also aim for greater specificity in phenotypic characterization in efforts to better understand *DYRK1A* haploinsufficiency as a unique clinical profile. Continued study of ASD-associated genes, including *DYRK1A*, will allow for improved understanding of ASD subtypes and inform future approaches to personalized treatment.

## Conclusions

*DYRK1A* haploinsufficiency results in a clinical phenotype which includes microcephaly, intellectual impairment, the presence of vision and motor difficulties, feeding difficulties, language delays, and ASD risk. The *DYRK1A* profile suggests a potential subtype of ASD. Despite a consistent profile, quantitative assessment highlights heterogeneity in the severity of impairments, with parental phenotype, reflecting genetic background, as a likely contributor to that variability among individuals.

## Additional files

Additional file 1: Variant information for known de novo *DYRK1A* mutation cases. Full variant information for previously published *DYRK1A* variants and patients seen at UW. Previously published cases identified by first author last name and UW cases denoted TXXX. Sheets organized by variant type: snvs/indels, cnvs, mosaic variants, and translocations/inversions. (XLSX 17 kb)

Additional file 2: Variant locations via UCSC Genome Browser. Presentation of *DYRK1A* isoforms and variant locations for previously published and UW cases. Figure generated in UCSC Genome Browser [54]. (PDF 201 kb)

## Abbreviations

ASD: Autism spectrum disorder
CHR: Chromosomal rearrangement (including CNVs, translocations, and inversions)
CNV: Copy number variation
Pub-CHR: Previously published chromosomal rearrangements
Pub-SNV: Previously published disruptive SNVs
SNV: Single nucleotide variant (disruptive variants include nonsense, frameshift, splice site, and missense mutations)
UW-SNV: UW study participants with disruptive SNVs to *DYRK1A*
