# Supplementary figures and images for: Clinical phenotype of ASD-associated DYRK1A haploinsufficiency
Source: Mol Autism. 2017 Oct 5;8:54. doi: 10.1186/s13229-017-0173-5 (PMC5629761; doi:10.1186/s13229-017-0173-5)

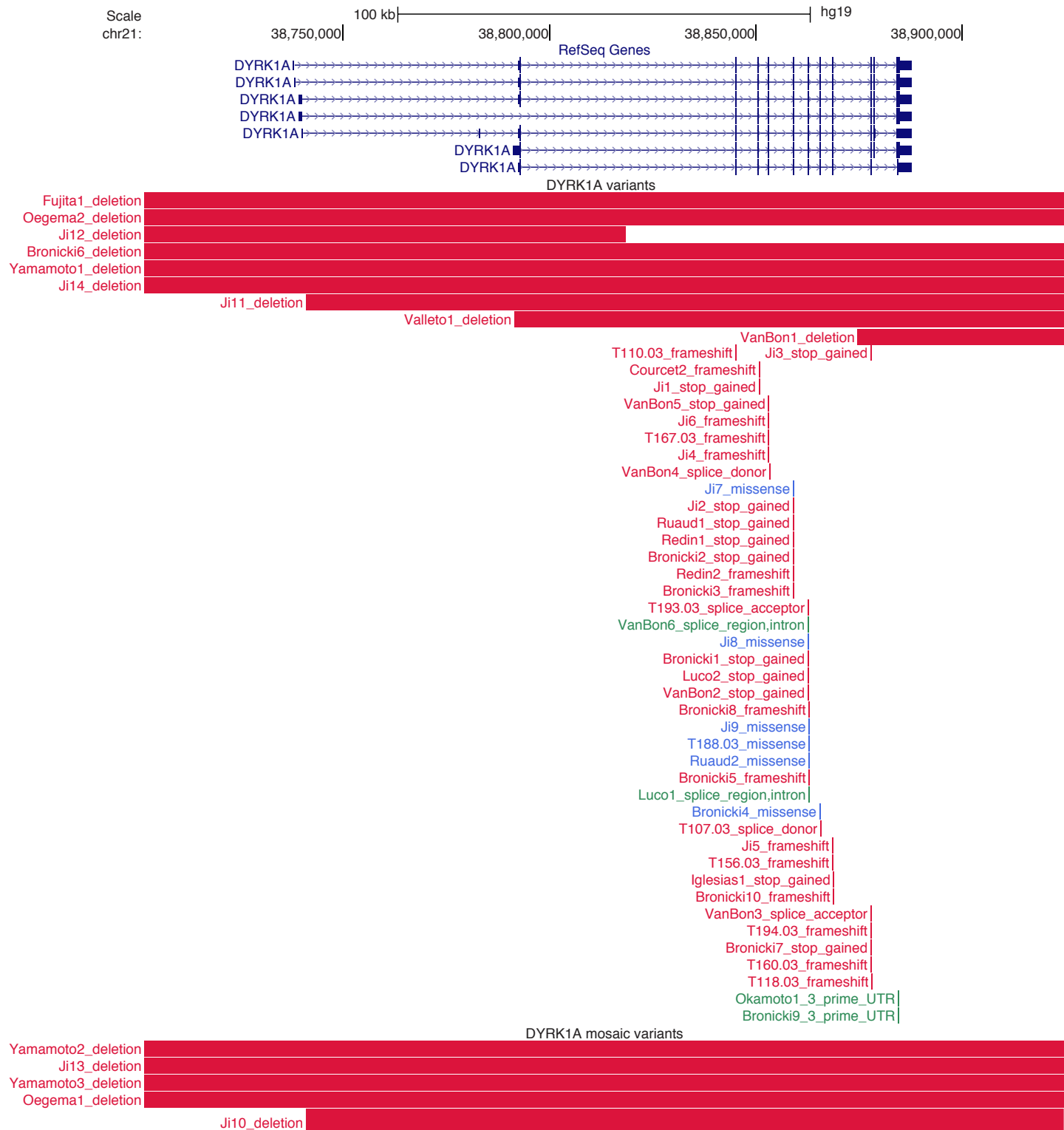

Supplement: Supplementary file 2 — Variant locations via UCSC Genome Browser. Presentation of DYRK1A isoforms and variant locations for previously published and UW cases. Figure generated in UCSC Genome Browser [54]. (PDF 201 kb) [file 13229_2017_173_MOESM2_ESM.pdf]
